# Facial Neuralgia

**Published:** 1844-05-04

**Authors:** 


FACIAL NEURALGIA.

Dr. Brooks, of the Cheltenham General Hospital, narrates a case of facial neuralgia occurring in a young lady, seventeen years of age, of a nervous hysterical temperament, in whom, after belladonna, veratrine, strychnia, and iodine, had been tried unavailingly, aconitine, topically employed, effected a cure. Two grains of the aconitine were dissolved in spirits of wine, and incorporated with two drachms of lard, of which a piece, the size of a pea, was rubbed into the face on the accession of the pain. The application of the ointment occasioned a twitching, stinging sensation of the skin, and after a few trials, the paroxyms were diminished in frequency and severity. It was used six times daily for about three weeks, and continued for some time afterwards, longer intervals being gradually allowed to elapse between each application. Dr. Brookes considers that if further experience confirms the opinion he entertains of this remedy, it will prove a valuable addition to the materia medica—*Ibid.*

